# Unraveling the Heterogeneity and Ontogeny of Dendritic Cells Using Single-Cell RNA Sequencing

**DOI:** 10.3389/fimmu.2021.711329

**Published:** 2021-09-09

**Authors:** Binyao Chen, Lei Zhu, Shizhao Yang, Wenru Su

**Affiliations:** State Key Laboratory of Ophthalmology, Zhongshan Ophthalmic Center, Sun Yat-Sen University, Guangzhou, China

**Keywords:** dendritic cells, single-cell RNA sequencing, cellular heterogeneity, the ontogeny of dendritic cells, tumor-infiltrating dendritic cells

## Abstract

Dendritic cells (DCs) play essential roles in innate and adaptive immunity and show high heterogeneity and intricate ontogeny. Advances in high-throughput sequencing technologies, particularly single-cell RNA sequencing (scRNA-seq), have improved the understanding of DC subsets. In this review, we discuss in detail the remarkable perspectives in DC reclassification and ontogeny as revealed by scRNA-seq. Moreover, the heterogeneity and multifunction of DCs during diseases as determined by scRNA-seq are described. Finally, we provide insights into the challenges and future trends in scRNA-seq technologies and DC research.

## Introduction

Dendritic cells (DCs) are the most efficient antigen-presenting cells and form essential interconnections between innate and adaptive immunity (1, 2). As innate immune components, DCs distinguish and monitor the pathogen‐ and danger‐associated signals and subsequently initiate acute immunological responses. During an adaptive immune response, DCs preprocess various extracellular and intracellular antigens and introduce them to naïve T cells *via* major histocompatibility complex molecules (3). Generally, DCs are the starting point of both innate and adaptive immune system responses.

The incredible heterogeneity and diversity of DCs are well matched to the precise and intricate functions of the immune system (4, 5). In humans, various extraneous threats prime the immune system to develop effective defensive responses. However, excessive immune responses can lead to the intolerance of autologous antigens. Therefore, an appropriate immune response requires the coexistence of multifarious types of DCs, with each subset trained to respond to specific pathogens and collaborate with relevant T cell subtypes. However, limited by technologies and methodologies, it remains challenging to determine a comprehensive DC subtype atlas and evaluate their biomarkers, cell lineage, and role in immune-related diseases (6, 7). Therefore, a new perspective and rational approaches to DC research are urgently needed.

Since the first report on single-cell transcriptome profiling in 2009 (8), single-cell RNA sequencing (scRNA-seq) technology has greatly advanced. Currently, the major available platforms are based on next-generation sequencing (9–15). Shortly after the evolution of this approach, third-generation sequencing technologies emerged, improving the limited read length of next-generation sequencing (16). The appearance and prevalence of scRNA-seq methods have revolutionized DC research, providing insights into DC heterogeneity and ontogeny. A comprehensive literature review focusing on the application of scRNA-seq in DC research is urgently desired by immunologists.

In this review, we discuss recent landmark findings in DC classification and the lineages determined using scRNA-seq technologies. We focus on the applications of scRNA-seq in pathological states, particularly in the tumor environment. Finally, we describe the challenges and future trends of scRNA-seq technologies and DC research.

## Strengths of scRNA-seq Technology

scRNA-seq involves the amplification and sequencing of the cell-specific transcriptome to generate a comprehensive gene expression atlas at single-cell resolution (17, 18). Although all body cells share nearly identical genotypes, each cell contains unique transcriptomic information based on its microenvironment and cell conditions. Bulk sequencing of millions of cells in collective samples yields averaged gene expression profiles of all cells and masks heterogeneity among cells (19, 20). Typically, because immune cells are highly heterogeneous and intricate, some subsets accounting for only a small percentage of the total cells are not detected by traditional methods. Hence, considering the sensitivity to minimal subsets, scRNA-seq provides more detailed results than bulk sequencing (21). Additionally, some of the more recent technologies, such as multicolor flow cytometry and cytometry by time-of-flight, can simultaneously measure over 50 protein markers per cell, providing relatively comprehensive insights into the immune proteome at the single-cell level (22, 23). However, scRNA-seq is an unbiased approach for automatically reclassifying immune subsets, which can overcome the limitations of existing methods and enable the identification of new cell types and states (24, 25).

Determining the myeloid developmental trajectory by traditional methods remains difficult. This limitation of bulk RNA sequencing is particularly magnified in studies of cells in dynamic development—for instance, the evolution of progenitor cells into their final differentiated populations through multiplex transitional phases is challenging to detect by bulk sequencing (26). Moreover, the classical hierarchical staged model of hematopoiesis is mostly based on subjectively purified cell populations, which were passively segmented into partitions while overlooking the discrepancies within gates as well as transition states between gates (27, 28). Because pre-set markers that define a cell population are not required and full-scale descriptions of transcriptomic profiling of rare, even transient, transcriptional states can be determined, scRNA-seq has promoted studies of the DC hierarchy.

DCs undergo differentiation, migrate, and respond to environmental stimuli, showing a wide heterogeneity in their markers and transcriptomic characteristics (4, 29). Diseases, particularly carcinogenesis, amplify this nonuniformity. In some cases, the small number of cells is considered responsible for the pathological state, which is difficult to detect (30, 31). By relying on prior knowledge of subpopulation markers in traditional flow cytometry and histology, the substates of disease-specific DCs may be ignored. The superior ability of scRNA-seq to comprehensively capture transcriptional signatures overcomes this challenge and has improved the understanding of the roles of DC substates during homeostasis and diseases.

## Applications of scRNA-seq in DC Studies

**Figure 1:**
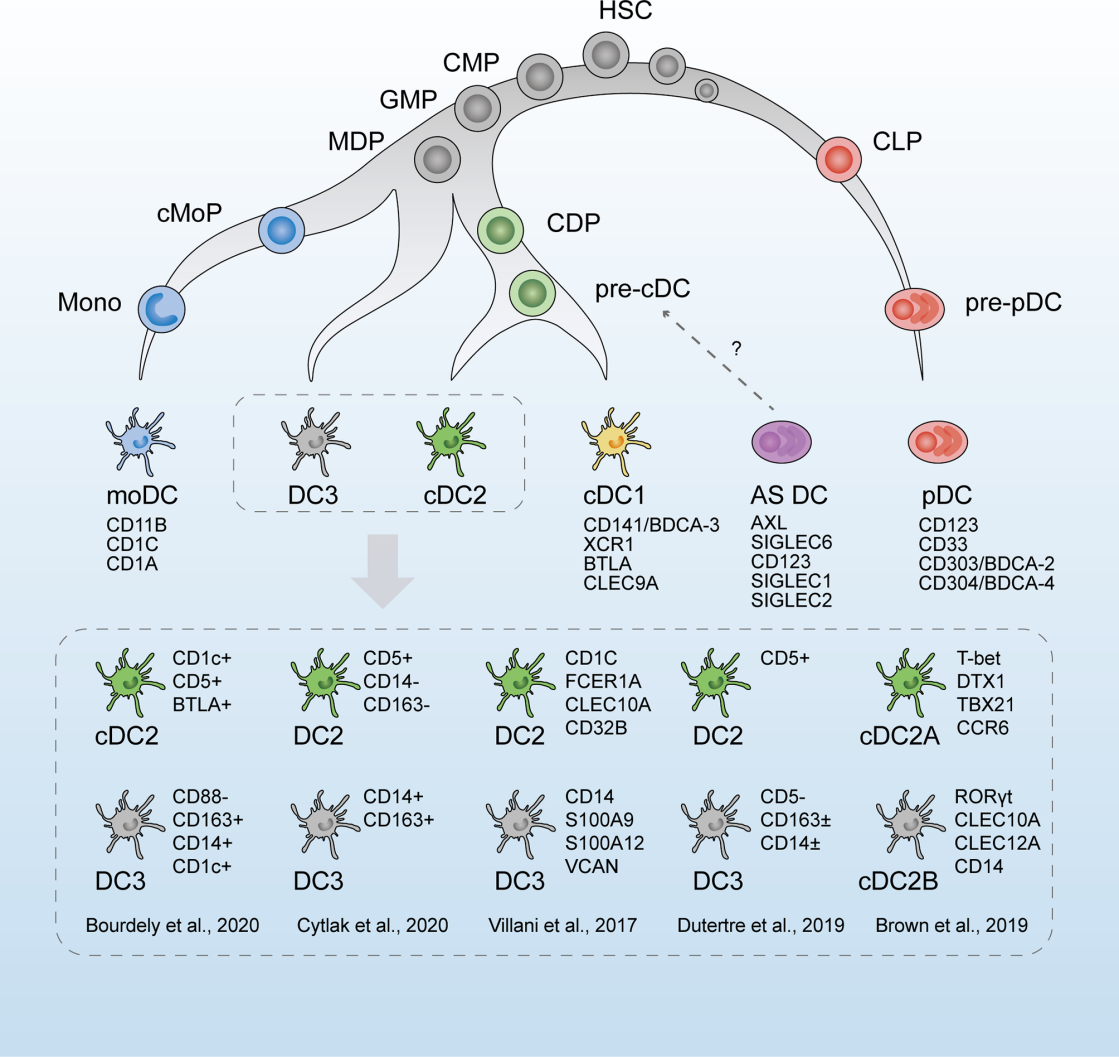
The revised developmental trajectory and classification of human dendritic cells (DCs). The revised developmental trajectory of the DC lineage is continuous, with progenitors already committed to different lineages at the early stages. The classic DC subsets (moDCs, cDC1s, cDC2s, and pDCs) are present. Previous homogenous subset cDC2s is considered heterogeneous, which is subdivided by different markers in scRNA-seq studies by Villani et al. (42), Brown et al. (43), and Dutertre et al. (44). Based on Bourdely et al. (45) and Cytlak et al. (46), DC3s are defined as a new subset, separately derived from MDPs. The origin of pDCs is revised as the lymphoid progenitors. Whether AS DCs (AXL^+^SIGLEC6^+^ DCs) are cDC precursors remains to be verified. HSC, hematopoietic stem cells; CMP, common myeloid progenitors; CLP, common lymphoid progenitors; GMP, granulocyte macrophage progenitors; MDP, monocyte–macrophage DC progenitors; CDP, common DC progenitors; cMoP, common monocyte progenitor; Mono, monocytes.

As the major antigen-presenting cells, DCs participate in both immunological defense and immune tolerance (32, 33). Functioning in both innate and adaptive immunity, DCs monitor infection sites and danger signals or permeate pathologic environments to take up tumor antigens (34). Upon activating naïve T cells, DCs simultaneously produce various cytokines and chemokines to regulate the immune response (35). Furthermore, DCs play an indispensable tolerogenic role by maintaining immune homeostasis and arresting the autoimmune response (33). The outcome of a rapid and precise immune response and balanced immune tolerance greatly relies on the phenotypic and functional heterogeneity of DCs. In the meanwhile, the complex immune microenvironment and immune response requirements remodel the DC subsets, jointly contributing to the intricate categorization and lineage development (36). Thus, establishing a comprehensive DC atlas is urgent for immunological researches and clinical applications. To this end, single-cell transcriptomics has been successfully used to identify new DC subsets, depict unbiased classifications, map cell lineages, and determine the pathological or protective role of DCs in diseases. Several beneficial reviews have been available on the application of scRNA-seq on myeloid cells (37–41). We discuss the landmark findings of DCs in health and disease as determined by scRNA-seq below (**Figure 1**).

### Revision of the Classification of DCs and Identification of New Subsets

The exploration of DC subsets dates from the observation that CD8 was expressed on some, but not all, DCs in mice (47). Polychromatic flow cytometry enabled the assessment of multiple cellular markers and initial exploration of DC phenotypic heterogeneity (48).

The DC classification proposed in 2014 was primarily based on ontogeny, followed by the function and phenotype (49). According to the previous view, the common DC progenitor (CDP)-derived DCs could be subdivided into three main subtypes: (i) type 1 conventional or classical DCs (cDC1s), (ii) type 2 conventional or classical DCs (cDC2s), and (iii) plasmacytoid DCs (pDCs) (49). It was believed that cDCs and pDCs, derived from bone marrow (BM) CDPs, underwent an intermediate stage known as pre-DCs. These precursors then migrated to the peripheral blood and tissues where they matured into cDCs and pDCs (50–53). Generally, CD11c^+^ cDCs are professional antigen-presenting cells that activate CD4^+^ and CD8^+^ T cells (54), whereas pDCs generate type I interferon (IFN) during viral infections. Additionally, monocyte-derived DCs (moDCs) are absent from homeostasis but are common at inflammatory sites (55).

Substantial heterogeneity, accompanied by limited cell numbers, further complicates full-scale DC subtype analysis. Previous studies have found that immune cell subsets, which were considered homogeneous populations defined by surface markers, contain highly heterogeneous multi-component cell types (56, 57). Shalek et al. (58) took the lead to employ scRNA-seq to reveal the heterogeneity of DCs, revealing previously unobserved variations among seemingly identical cells. scRNA-seq has been extensively utilized to decipher the heterogeneous cell groups because of its unbiased approach and high sensitivity for detecting rare cell populations, which would otherwise be neglected by traditional approaches (59, 60). Compared with flow cytometry, single-cell transcriptomic techniques facilitate cell type identification and eliminate the requirement for known surface markers. It is, however, absolutely critical to realize, when interpreting clustered scRNA-seq data, that the technology on its own is unable to discriminate bona fide cell subsets from heterogeneous cell states which belong to the same cell lineage. Identification of new bona fide cell subsets requires additional experimental validation.

A newly proposed classification of DCs dependent on scRNA-seq by Villani et al. (42) in 2017 has led to a new perspective in DC research, which is widely referenced and discussed. Following scRNA-seq analysis and unbiased transcriptomic classification, six main DC groups were identified using a bioinformatic clustering approach and were named DC1–6. Further functional and phenotypic analyses confirmed this unsupervised sorting strategy, thus validating the respective physiological roles of the assumed DC subsets. DC1s analogous to the classic CD141^+^ cDC1s in humans were discriminated by the surface marker CLEC9A. DC2s and DC3s were two subtypes within CD1c^+^ cDC2s, the former of which was characterized by a major histocompatibility complex II-like gene set and the latter by a CD14^+^ monocyte-like gene set. Comparing the two clusters, DC3s strongly expressed an inflammatory gene program represented by CD14, S100A9, and S100A8. DC4s were represented as CD1c^-^CD141^-^cDCs, and literature regarding these cells is rare (61). DC5 (or “AS DCs”) was described as a new DC population defined by the expression of AXL, SIGLEC1, and SIGLEC6 markers. The gene expression signatures of AS DCs covered a spectrum between cDC2-like and pDC-like gene sets, suggesting a relationship to both pDCs and cDC2s cells. Classic pDCs were named DC6s.

The real identity of AS DCs remains controversial. This newly discovered DC population was reported to occupy 2 to 3% of human blood DC populations (42), and another scRNA-seq study verified their existence in human cord blood (62). Considerable heterogeneity is observed in AS DCs, as they are captured in both traditional pDC and cDC gates by flow cytometry, and their gene expression profiles cover the spectrum between cDC2-like (*e*.*g*., IFI30, ITGAX, LY86, GLIPR20, FGR, LYZ, and ENTPD1) and pDC-like (*e*.*g*., IL3RA, IGJ, NRP1, MZB1) gene sets. Villani et al. (42) verified the morphological and functional similarity of AS DCs with cDCs and observed their differentiation towards CD1c^+^ DCs *in vitro*. Several scRNA-seq studies supported similar populations as cDC precursors (44, 46, 63). Similarly, Lukowski et al. (64) identified a cluster of Sox4^+^ cDCs in the murine spleen, displaying a continuum of cDC and pDC lineage-mixed gene signatures, which had a similar transcriptional profile with AS DCs in humans. This population expressed elevated levels of pre-DC features (such as CX3CR1, FLT3, CD33, and CSF1R) and was hypothesized to represent a cell state of pre-DC to cDC transition. Alcántara-Hernández et al. (65) argued that AS DCs were lacking in human skin but detectable in lymphoid tissues, in line with observations for pDCs. In addition, they verified that “pure” pDCs (without AS DCs) activated *in vitro* could increase their antigen presentation potential and convert towards the cDC-like phenotype (upregulated HLA-DR, CD80, and CD11c). Furthermore, they also identified the murine equivalent of AS DCs and named these “transitional DCs” because of the continuum of pDC and cDC characteristics. This XCR1^-^CD11b^-^SiglecH^+^CX3CR^hi^ DC population shares major similarities with pDCs but inefficiently produces type I IFN (66). Before the identification of this presumptive AS DC population, similar undefined subsets with functional properties incompatible with their phenotype were discovered by flow cytometry, such as a CX3CR1^+^CD8α^+^ DC subset in the mouse spleen (belonging to cDCs) with pDC-like functions and transcriptome (67), as well as a CD2^hi^ pDC group that can secrete type I IFN and efficiently trigger T cell proliferation (68) and a CD2^hi^CD5^+^CD81^+^ pDC group in human blood, BM, and tonsil that could stimulate T and B cell activation but hardly produces type I IFN (69). Notably, this DC population has a CD2^+^CD5^+^ and pDC-like expression profile in common with AS DCs, which might represent two overlapping clusters (69). Whether these DC populations are homologs with diverse phenotypes in their respective microenvironment and developmental phases remains unclear. Additional research on the developmental trajectory of AS DCs would contribute to validating the identity of AS DCs.

Recent studies by scRNA-seq have expanded the understanding of the heterogeneity of cDC2s. According to Villani et al. (42), CD1c^+^ DCs were discrepant in the gene signatures, as DC3s expressing CD14 and “monocyte-like” gene signature were characterized by acute and chronic inflammatory genes, whereas DC2s were more similar to cDC1s. Later, in 2019, Dutertre et al. (44) proposed this CD14^+^CD1c^+^ DCs as a subpopulation of cDC2s. They identified CD5^+^ cDC2s to correspond to DC2s and CD5^-^ cDC2s to DC3s by single-cell protein and RNA analysis. This demarcation by CD5 is in line with the results of Yin et al. (70) and Korenfeld et al. (71). Similarly, in murine splenic DCs, two subtypes were divided within cDC2s by scRNA-seq, which were characterized by a mutually exclusive expression of T-bet and RORγt, with divergent pro- and anti-inflammatory roles *in vivo* (43). However, RORγt^+^ cDC2s were absent from human blood, and the CD1c^+^CLEC10A^+^ cDC2s (DC2,3s) corresponded to T-bet^+^ cDC2s. An analog to the mouse RORγt^+^ cDC2s subset was found in the human spleen and defined as CD1c^lo^CLEC10A^–^CLEC4A^hi^ cDC2s (43). In addition to the intrinsic heterogeneity of cDC2 subpopulations, interindividual variation was observed by scRNA-seq. The phenotypic profiles and subset frequencies of cDC2s varied dramatically among individuals, unlike the other subsets (65). This discrepancy between species and tissues further complicated the understanding of the cDC2 family.

There remained disputes on whether these putative DC3s belonged to monocytes or cDC2s or just represented a bona fide DC lineage independent of cDCs or pDCs (72). moDCs are the most abundant DC subset at the inflammatory site and arise from recruited monocytes (73). Harboring monocyte-like gene profiles, DC3s were once assigned to moDCs. A circulating CD1c^+^CD14^+^CD163^+^ cDC2 subset, related to the DC3s, was found to expand correlatively with disease activity in patients with systemic lupus erythematosus (SLE). These cells exhibited a pro-inflammatory transcriptomic profile and secreted pro-inflammatory mediators that might contribute to SLE physiopathology (44). Bourdely et al. (45) proposed circulating DC3s as immediate precursors of resident inflammatory DCs, and cells with DC3 phenotype were found to infiltrate breast cancer tissues and correlate positively with resident memory T cells. The overlap of their gene signature and functional ability seems to equate DC3s and moDCs. As monocyte derivation is the salient feature of moDCs, the ontogeny of DC3s should be the most convincing evidence of whether these two components are equivalent. As shown by scRNA-seq (45), DC3s developed directly *via* a DC3-restricted progenitor contained in early granulocyte–monocyte and DC progenitors, distinct from cDC-restricted CDPs or monocytes. In addition, GM-CSF alone, but not FLT3L, efficiently supported the differentiation of DC3s, in contrast to traditionally defined cDCs. Similarly, Cytlak et al. (46) demonstrated that DC3s developed from granulocyte–monocyte progenitors along an IRF8^low^ trajectory, separate from pDCs, cDC1s, or cDC2s. These scRNA-seq studies supported DC3 as a bona fide DC lineage, independent of cDCs, pDCs, or monocytes (74).

Notably, Calzetti et al. (75) pointed out that DC4s corresponded to a group of CD14^dim/-^ CD16 ^++^ monocytes by using a gating strategy based on the lack of CD14 expression only; thus, these cells could not be considered as a newly discovered DC population. This finding agrees with that of Dutertre et al. (44), who found that signature genes and markers of DC4s showed the highest expression in CD16^+^ monocytes but were undetectable in any DCs.

The scRNA-seq cluster-based extension of DC diversity represents a breakthrough in the traditional cognition of DCs and has significantly broadened our understanding of these cells. However, newly identified DC populations remain to be validated by ontogenic and functional verifications, which we discuss hereinafter.

### Trajectory Analysis of DC Lineages

Inferring the lineage trajectory based on transcriptomic profiles of individual cells is a promising application of scRNA-seq analysis (76, 77). From the static snapshot data of a population of cells at different stages in a developmental process, the pseudotime trajectory analysis calculates and reconstructs the dynamic differentiation processes. Multiple algorithms have been exploited to achieve the single-cell pseudotime trajectory analysis, including Monocle 2 (78), Monocle 3 (76), Slingshot (79), TSCAN (80), and others. No single method works for all the datasets, and the best practice depends on the structure of the trajectory and the sample size of the dataset. Of note is that, in pseudotime inference, cellular trajectories are determined purely by their transcriptional profiles, which, however, might not completely reflect the cell states (81). Indeed the bifurcations in gene expressions are thought to lag behind the actual fate decisions (82). Hence, it is recommended to combine single-cell transcriptomics with other cellular aspects, such as chromatin state, spatial arrangement, and phosphorylation, to validate the inferred cell transitions and developmental trajectory (83, 84).

A recently introduced concept, RNA velocity, has been developed to infer developmental trajectories in scRNA-seq data (85). Based on the ratio of spliced (mature) and unspliced (nascent) mRNA of an individual gene at a given time point, the positive or negative change in mRNA abundance, namely, RNA velocity, can be used to indicate the future states of cells (86). Velocyto, proposed by La Manno et al., is a potent model to estimate RNA velocity using scRNA-seq (85). More recently, the newly developed method, scVelo (86), breaks the central assumptions of a common splicing rate in RNA velocity and largely shortens the time and lessens the memory consumption than that of Velocyto by solving the full transcriptional dynamics of splicing kinetics using a likelihood-based dynamical model.

According to the previous view, the developmental trajectory of hematopoietic lineage was accepted as stepwise and staged (87, 88). In the BM, the myeloid lineage stemmed from common myeloid progenitors to granulocyte–macrophage progenitors and then transformed into monocyte–macrophage DC progenitors (MDPs). DC-committed precursor cells, known as CDPs, continued. CDPs served as progenitors for pDCs and pre-cDCs. MDP-derived monocytes and CDP-derived pDCs, pre-cDCs, migrated into peripheral tissues through the bloodstream, where monocytes differentiated into moDCs and pre-cDCs into two classical DC subtypes, cDC1s and cDC2s (89).

ScRNA-seq technologies have challenged the previous understandings of the ontogeny of hematopoiesis (90–92). One of the most important notions is that the hematopoietic system is less of a stepwise process than previously suggested. In contrast to the traditional concept of hierarchy deferring to multi-, oligo-, and unipotent progenitors, there is high cell-to-cell variability in the propensity of hematopoietic progenitors to differentiate into separate lineages (93, 94)—for instance, in addition to the reported heterogeneity among CDP populations with distinct differentiation priming towards cDCs and pDCs, Schlitzer et al. (95) focused on the potential differentiation potency towards cDC1s and cDC2s among murine CDPs. Previous studies showed that single CDPs cultured *in vitro* differentiate preferentially into the cDC1 or cDC2 subsets (50). Single-cell transcriptomic analysis validated that the biased gene expression sets towards cDC1 and cDC2 lineages became visible at the CDP stage in the BM (95). Notably, the transcriptomic signatures overlapped among putative MDP, CDP, and pre-cDC subsets. This finding indicated that cells in the MDP, CDP, or pre-cDC pool had already transformed into the next differentiation stage at the transcriptome level despite the preservation of their protein signatures. Similarly, Bagadia et al. (96) identified the earliest committed cDC1 progenitors within CDPs. This is also the case for pre-cDCs, which already contain committed pre-cDC1s and pre-cDC2s (63).

In contrast to the classical “ball-and-stick” model in which all cells within a population behave equally, Naik et al. (97) proposed a revised continuous model of DC differentiation, where early progenitors have already committed to different lineages. In addition, the “Autobahn” model is introduced by Bassler et al., in which progenitors harbor early cell fate commitment and also multipotent plasticity as an adaptation to environmental changes (39). Two models both highlight the continuum of hierarchical differentiation spanning multiple intermediate states as well as the pre-commitment of progenitors. Comparatively, the Autobahn model stresses more on the changeability of lineage fate to fit in various environmental changes. Lin et al. (98) found that the FLT3 ligand-mediated increase of cDC1 output was achieved by the selective expansion of cDC1-primed progenitors, without comprising other lineages. This observation contradicts the changeable fate mapping of progenitors under external stimuli in the Autobahn model.

pDCs are considered to vary from cDCs in terms of derivation, function, and surface markers (53). The ontogeny of pDCs appeared to be controversial and promiscuous, as contradictory evidence was reported for both a myeloid and lymphoid origin of pDCs (51, 99, 100). Previously, pDCs were classified within the myeloid compartment, but scRNA-seq studies have questioned this hypothesis (101). Herman et al. (102) revealed a common precursor cluster shared by B cells and pDCs, indicating the lymphoid origin of pDCs. Verification was performed by assessing the differentiation potential of sorted precursors. Similarly, Rodrigues et al. (103) proved that pDCs developed mostly from Ly6D^+^SiglecH^+^IL-7R^+^ lymphoid progenitor cells and showed transcriptional and functional heterogeneity from myeloid-derived “pDC-like” cells. Subsequently, in 2019, Dress et al. (104) proposed that pDCs, developed from Ly6D^+^CD81^+^ lymphoid progenitors in mice, were completely independent of the myeloid DC lineage. A marked proportion of common lymphoid progenitors harbored a characteristic gene program, which denoted the earliest detected pDC priming. Additionally, they identified the newly discovered pDC-primed Ly6D^+^ pDC progenitors in every conventionally defined cDC progenitor population, which may account for the inconsistent observations of pDC derivation.

The origin of moDCs and whether the origin depends on environmental factors are other important issues being addressed by many groups. A study involving scRNA-seq showed that the moDC-primed differentiation potency already existed in a separate subset of Ly6C^+^ monocytes in the murine BM (105). However, an opposing hypothesis was also presented: all human blood CD14^+^ monocytes are potential moDCs producers, in the case of certain cytokines in conjunction with environmental ligands of the aryl hydrocarbon receptor (106). Consistently, Mildner et al. (107) demonstrated that murine Ly6C^+^ and Ly6C^-^ monocytes are homogeneous populations according to scRNA-seq.

In summary, scRNA-seq has improved the understanding of the origins and trajectory of DC subset differentiation, identity, and development. Although interesting, some contradictory findings require further exploration.

### Application of scRNA-seq in Disease-Related DCs

Tumor-infiltrating myeloid cells (TIM) comprise various immune subsets that could profoundly shape cancer development (108). By directly interacting with malignant cells or indirectly releasing cytokines and chemokines, TIM could condition anti-tumoral protection and also take part in immune surveillance and favor tumor progression under certain circumstances (109). DCs account for a small fraction of TIM but play a key role in presenting tumor antigens and priming T cells (110).

**Figure 2:**
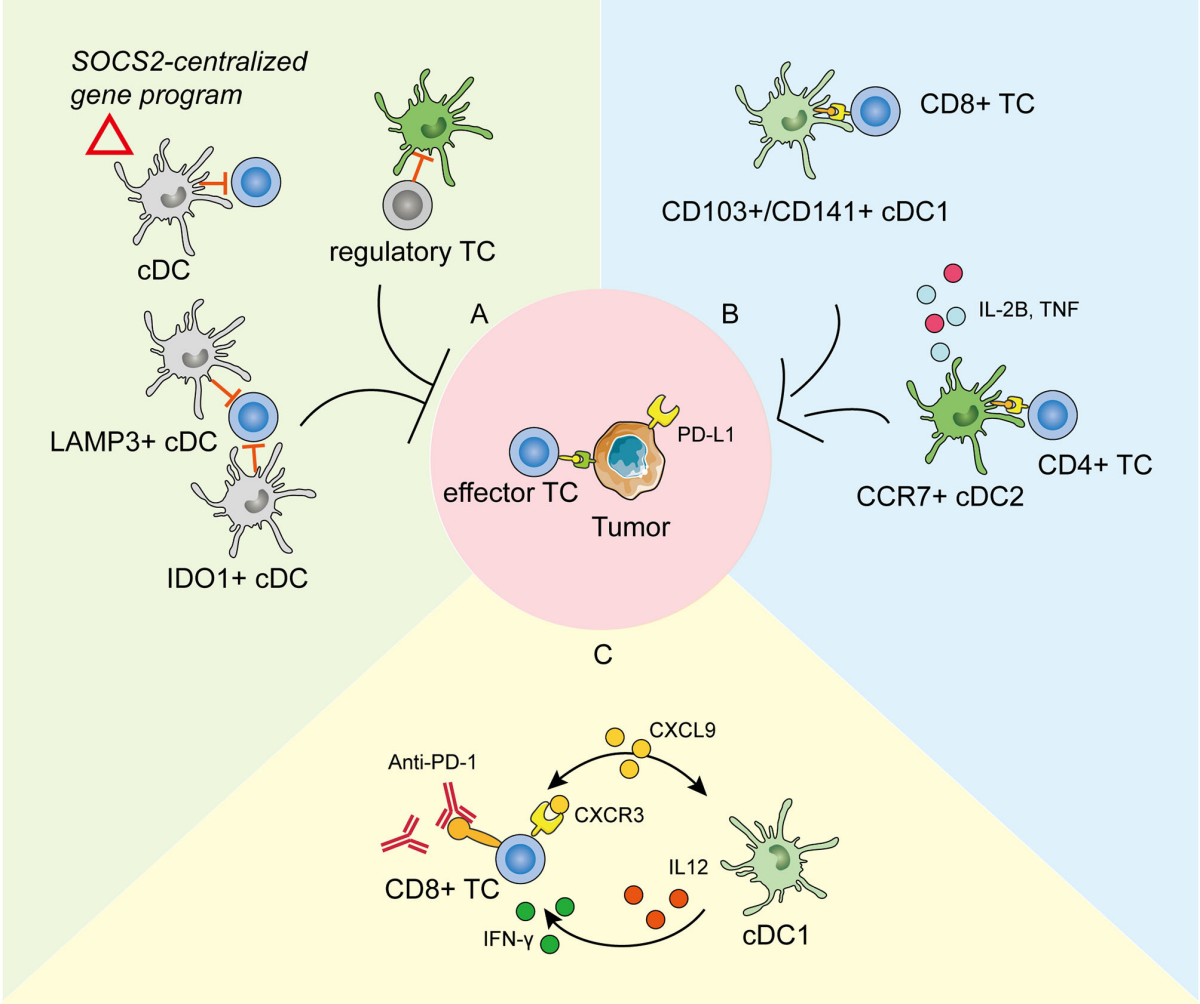
The roles of tumor-infiltrating dendritic cells (DCs) in tumor development and anti-PD-1 immunotherapy as revealed by scRNA-seq. **(A)** DCs in the tumor environment could be modified to repress the anti-tumoral immunity and promote cancer development in different ways. cDCs expressing the SOCS2-centralized gene program could facilitate tumor immune surveillance (114). Clusters of IDO1^+^ cDCs and LAMP3^+^ cDCs are reported to repress the proliferation and function of effector T cells (115, 116). DCs could also be restrained by regulatory T cells (117). **(B)** cDC1s take in the tumor antigens and activate effector CD8^+^ T cells (TC). cDC2s activate the CD4^+^ TCs and secrete pro-inflammatory cytokines to promote anti-tumoral immunity. **(C)** The crosstalk between cDC1s and CD8^+^ TCs through chemokines and cytokines is pivotal in response to effective anti-PD-1 immunotherapy.

In cancer, cDC1s form the predominant DC population and are specialized to take in the tumor antigens and deliver them to the lymph nodes (LNs); therein, they activate anti-tumoral T cells by cross-presentation (111). In terms of cDC2s, it is considered that they activate CD4^+^ T cell and Th17 cells but do not deliver antigens to LNs (112). moDCs derived from circulating monocytes can also participate in the tumor microenvironment and function similarly to cDC1s. pDCs uniquely produce pro-inflammatory IFN to promote DC-mediated anti-tumor responses (113). Therefore, deciphering a comprehensive transcriptomic profile of tumor-infiltrating DCs by scRNA-seq has greatly promoted the knowledge of tumor immunity and provided potential targets for anti-tumoral therapy (**Figure 2**).

Generally, the single-cell transcriptomes of DCs in the tumor environment present dynamic alterations during tumor progression (118). Moreover, tumor-infiltrating DCs exhibit unique transcriptional profiles compared with peripheral blood DCs, along with interior heterogeneity (119). Across species, DCs maintain largely conserved, as shown by scRNA-seq of TIM in mice and humans (119, 120). These provide valuable evidence for linking the experimental results in mice with the clinical outcomes and therapeutic responses in humans as well as unfavorable evidence for speculating tumor-infiltrating DCs based on peripheral blood.

Previous studies have reported that tumor-infiltrating cDC1s could effectively enhance effector T cells and protective responses and correlate with a favorable prognosis in several cancers (121–123). By scRNA-seq of different degrees of precursor lesions of pancreatic ductal adenocarcinoma, Bernard et al. (118) found that the proinflammatory immune component in the tumor environment, including activated DCs and cytotoxic T cells, was progressively depleted during neoplastic progression. Accordingly, recent single-cell profiling of early lung adenocarcinoma revealed that human cDC1s were significantly scarce at the tumor site compared to that at non-tumor lesions, which might account for the enrichment of non-functional T cells in tumor lesions (124).

scRNA-seq helped to (117) identify the specialized role and unique function of cDC2s in tumor immunology. Profiling of tumor-draining LNs of mice showed that migratory cDC2s expressing CCR7 were responsible for initiating CD4^+^ T cell priming, but not differentiation, leading to defective anti-tumoral effector T cells. The therapeutic depletion of regulatory T cells effectively enhanced cDC2 migration and reverted their phenotypical dysfunction, thus enabling productive anti-tumoral CD4^+^ T cell priming in the LNs (117). This study revealed that the balance between cDC2s and regulatory T cells determined the protective antitumor effect of CD4^+^ T cells. Aside from antigen presentation, a tumor-enriched cDC2 cluster was reported to highly express pro-inflammatory mediators such as interleukin-1B and tumor necrosis factor (125), which may resemble the reported circulating pro-inflammatory DC3s (42, 44, 45). In line with the anti-tumoral role, a recent scRNA-seq study suggested that the expression of CD207, the characteristic marker of cDC2s, correlated positively with the survival of patients with lung cancer (119).

Except for the anti-tumorigenic roles, DCs can also be modified to actively support cancerogenesis and promote immune escape (126). Normally, mononuclear phagocytes maintain a shared gene program during differentiation and entry into healthy tissues to keep the immune homeostasis (127). However, in some cases, this conserved physiological homeostatic program expressed by migratory DCs might be made use of by tumor-immune surveillance. Single-cell transcriptome profiles of human melanoma metastases suggested that enrichment of this homeostatic signature in DCs functioned in tumor immune escape, which was IFN γ dependent and SOCS2 (suppressor-of-cytokine-2) centralized (114). Depletion of SOCS2 restored anti-tumoral immunity by expanding DC-priming T cell immunity. In addition, a cluster of IDO1^+^ cDCs was identified in tumor environments and was predicted to impair T cell proliferation and cytotoxicity by promoting tryptophan depletion and kynurenine production (115). Interestingly, a cluster of tolerogenic and regulatory DCs, highly expressing migration (CCR7)- and maturation (LAMP3)-related genes, along with various chemokine ligands (CCL17, CCL19, and CCL22), was presumably able to interact with regulatory T cells and exhausted CD8^+^ T cells by binding to corresponding receptors in nasopharyngeal carcinoma at single-cell resolution (116). This immune-suppressive transcriptomic pattern in LAMP3^+^ DCs has been widely detected in various tumors by scRNA-seq, including in bladder urothelial carcinoma (128), hepatocellular carcinoma (129, 130), and lung cancer (119, 131), suggesting that universal crosstalk fosters an immune-suppressive niche for the tumor microenvironment. A pan-cancer single-cell immune atlas covering 15 cancer types verified the broad presence of LAMP3^+^ DCs, with variable abundance in different cancers (125). Furthermore, AXL and IL-4 signaling partially drive this immunoregulatory program, and IL-4 blocking efficiently rescued the immune depression, providing an antitumor therapy target (131). Zheng et al. (132) added the higher expression of specific transcription factors (including RELB, IRF1, FOXO1, and ETS1) to the features of LAMP3^+^ DCs in human esophageal squamous cell carcinoma. To further explore their lineage origins, studies have suggested that the tumor-infiltrating LAMP3^+^ DCs could arise from both cDC1s and cDC2 (129, 131). Notably, these differently originated LAMP3^+^ DCs maintained specific transcriptomic properties, were regulated by different ligand–receptor pairs, and might perform diverse functions (125). Therefore, as revealed by scRNA-seq, tumor development is accompanied by the transition or differentiation of functional cDCs into a regulatory phenotype or functional defectiveness to create an immunosuppressive milieu.

The crosstalk between tumor-infiltrating DCs and T cells plays an important role in cancer immunotherapy (133, 134). Immune checkpoint blockade (ICB) has emerged as a promising method in cancer treatment (135). Despite considerable clinical responses received, scRNA-seq has promoted the mechanism study to extend the benefits to resistant tumors. Previous studies have demonstrated that the expansion and activation of tumor-infiltrating cDC1s could enhance the therapeutic effect of the PD-1 blockade (110, 122). Based on scRNA-seq analysis on experimental melanoma mouse models, Lelliott et al. (136) showed that combined BRAF, MEK, and CD4/6 inhibition triple therapy could result in the marked depletion of cDC1s from the tumor milieu, the absence of which might contribute to non-response to ICB and poor survival in patients with melanoma. Moreover, the mechanisms of how tumor-infiltrating DCs influence immunotherapy efficiency have become clearer by scRNA-seq. Garris et al. (137) showed that the tumor-infiltrating DC-derived IL-12 was indispensable in effective anti-PD-1 response. Meanwhile, the IFN-γ released by activated CD8+ T cells further activated cDC1s. In line with this, scRNA-seq analysis on a mouse colorectal cancer model MC38 suggested that anti-CD40 agonist specifically activated a subpopulation of cDC1, leading to the upregulation of IL-12, which could enhance Th1 development and IFN-γ production by CD8^+^ T cells (120). Chow et al. likewise presented that the cellular crosstalk mediated by cDC1s-derived CXCL9 and CXCR3 on T cells was pivotal for the proliferation and function of intratumoral CD8^+^ T cells in response to anti-PD-1 treatment. Apart from cDCs in tumor immunity, the scRNA-seq of human melanoma biopsies suggested that moDCs correlated with PD-1 responsiveness and effector T cell activity, and more importantly, targeting moDCs by anti-CD40 antibody could enhanced PD-1 ICB efficacy (138).

As antigen-presenting cells, DCs can influence autoimmunity and infection in a complex, even in a bidirectional, manner, as they can promote either immune tolerance or prime T cell differentiation—for instance, moDCs, also described as inflammatory DCs, have been reported to dominate in various inflammations (139). Janela et al. (140) identified the exact dominant DC population during cutaneous bacterial infection and related pathways. They revealed that a minor cell group of activated EpCAM^+^CD59^+^Ly-6D^+^ cDC1s controls neutrophil recruitment to the inflamed site and survival and function by secreting the cytokine vascular endothelial growth factor-α. Dutertre et al. (44, 140) identified a distinct subset of CD5^-^CD163^+^CD14^+^ cDC2s [DC3s in (42)] as circulating inflammatory DCs. The proliferation of inflammatory CD163^+^CD14^+^ DC3s was positively correlated with the severity of SLE. In terms of neuroinflammation, Jordão et al. (141) proposed that, although DCs were scarce in the homeostatic central nervous system, their density highly increased during neuroinflammation. DCs and monocyte-derived cells exhibited a high diversity during experimental autoimmune encephalomyelitis and played major roles in antigen presentation to initiate the disease pathology. Martini et al. (142) used scRNA-seq to map the cardiac immune composition in a standard murine nonischemic, pressure-overload heart failure model and found that the relative abundance of DCs decreased in the early and late disease stages. Furthermore, the DC cluster was not homogeneous but could be subdivided into two subclusters, the larger one of which is transcriptionally active and associated with DC differentiation. In a bleomycin-induced pulmonary fibrosis mouse model, Peyser et al. (143) observed a significant increase in the DC population compared to saline-treated lungs. The role of increased DCs in pathogenesis merits a closer examination. Additionally, DCs have been reported to engage in diverse pathogenic and protective mechanisms during atherogenesis (144). Cochain et al. (145) found a cluster of moDCs as the major atherosclerosis-associated cell population, representing 14.9% of the total CD45^+^ population in the atherosclerotic aorta.

Collectively, transcriptomic studies at the single-cell level can help to understand the phenotypic and functional profiles of DCs in the pathological state and predict the protective or stimulative role in disease development, thereby providing potential targets for treating immune-related diseases.

## Challenges and Future Perspectives

In the past 10 years, the application of scRNA-seq technologies has revolutionized the understanding of DC evolution, differentiation, and heterogeneity in homeostatic and pathological states. The historical delineation of cell populations based on known surface markers has been insufficient for comprehensively discriminating subsets, particularly concerning variable pathological conditions. In contrast, unsupervised analysis based on single-cell resolution transcriptomes performs better than conventional flow cytometry and bulk transcriptomic analysis using a minimal set of pre-selected markers.

However, as scRNA-seq captures a snapshot of transcriptomic profiles and DCs are dynamic cells, this approach is insufficient to define a bona fide DC subset or lineage as opposed to a transitory cell state of the same lineage. As the gene expression signatures of DCs are changing dynamically, responding to environmental stimuli and functional requirements, scRNA-seq-derived cell clusters based on differential transcriptomic gene expression should not be confused with the identification of bona fide cell subsets, but clusters can represent a cell subset or a cell state. This cell state can be related to the cell cycle of the cell, metabolic activity, ontogeny, maturation, or the microenvironment in which it resides. As mentioned above, the same population, cDC2, might be described and interpreted in divergent manners by different researchers. Hence, for a new cell type to be accepted beyond the precondition of discrepant transcriptomic programs, complementary functional and ontogenic evidence, that is, separate developmental pathways controlled by specific transcription factors, is necessary to determine a scRNA-seq-defined subset.

In addition, the challenges of defining subsets also lie in technologies and algorithms (146). Clustering is the key step in defining cell types based on the transcriptome. Despite unsupervised computational methods, the specific cluster resolution is set manually, which determines how many clusters are divided in the dataset. Computational methods aiding the selection of clustering resolution exist, but a final judgment and decision from the user are required (147). Under-clustering can hide a rare but biologically relevant population. By contrast, over-clustering can result in partitioning a population into several clusters that simply represent stochastic variations instead of biological states. Due to the low abundance of RNA captured from single cells, “dropouts” (zero reads detected in some cells but with a relatively high expression in others) are inevitably more common in scRNA-seq than in bulk sequencing, thus reducing the reliability of the results. Some imputation methods are developed to reduce the dropout effects before in-depth analysis (148, 149), but this inference still relies on information from the local cell community.

One promising future direction for scRNA-seq is the integration of the DC atlas across different species. A better understanding of the counterparts of DC subsets in humans and mice will lead to the development of potential clinical benefits. Moreover, single-cell multi-omics studies have received increasing research attention. Thus far, transcriptomes, methylomes, proteomes, and epigenomes from the same cell have been obtained and analyzed. Further studies will facilitate improvements in single-cell metabolomics and single-cell proteomes as well as integration of multi-omics single-cell analysis. scRNA-seq may be useful for mapping the DC atlas across species through an accurate and unbiased classification of subsets, and the intricate ontogeny of development and metabolomics, epigenomics, and proteomics can reveal how DCs function in physiological and pathological conditions.

## Author Contributions

WS contributed to the conception and design of this study. BC, LZ, and SY contributed to the drafting and revising of the article. WS gave final approval. All authors contributed to the article and approved the submitted version.

## Funding

This study was supported by the National Key Research and Development Program of China (2017YFA0105804).

## Conflict of Interest

The authors declare that the research was conducted in the absence of any commercial or financial relationships that could be construed as a potential conflict of interest.

## Publisher’s Note

All claims expressed in this article are solely those of the authors and do not necessarily represent those of their affiliated organizations, or those of the publisher, the editors and the reviewers. Any product that may be evaluated in this article, or claim that may be made by its manufacturer, is not guaranteed or endorsed by the publisher.
